# Differential Gene Expression of Efflux Pumps and Porins in Clinical Isolates of MDR *Acinetobacter baumannii*

**DOI:** 10.3390/life12030419

**Published:** 2022-03-14

**Authors:** Khalid I. AlQumaizi, Sunil Kumar, Razique Anwer, Shoeb Mustafa

**Affiliations:** 1 Department of Family Medicine, College of Medicine, AlMareefa University, Riyadh 11597, Saudi Arabia; kqumaizi@mcst.edu.sa; 2 Department of Medical Microbiology, Post Graduate Institute of Medical Education & Research, Sector-12, Chandigarh 160012, India; 3 Department of Biotechnology, Maharishi Markandeshwar (Deemed to Be) University, Mullana, Ambala 133207, Haryana, India; 4 Department of Pathology, College of Medicine, Imam Mohammad Ibn Saud Islamic University (IMSIU), Riyadh 13317-4233, Saudi Arabia; smahmad@imamu.edu.sa

**Keywords:** *Acinetobacter baumannii*, efflux pumps, gene expression, multidrug resistance, RT-PCR

## Abstract

Background: *Acinetobacter baumannii* is an opportunistic pathogen associated with healthcare infections and high mortality rates in intensive care units all over the globe. Porins and efflux pumps over-expression have been reported as contributing factors in escalating drug resistance and rendering treatment ineffective. In this study, we investigated the mechanisms of multidrug resistance (MDR) in *A. baumannii* clinical isolates. Methods: A total of 30 *A. baumannii* isolates were included in the present study from Nehru Hospital (PGIMER-Chandigarh) located in North India. Kirby Bauer disk diffusion assay and MIC were performed to determine the antimicrobial susceptibility pattern. Screening of beta-lactamases was performed using PCR. Relative gene expression of four RND, one MATE efflux pump, and two outer membrane proteins were determined using RT-PCR. Molecular typing of 22 isolates was carried out using MLST Oxford scheme. Results: *CarO* porin genes showed over-expression in 63% isolates followed by adeGandabeM efflux pump downregulation/underexpression (<0.5 fold), suggesting the carbapenem-susceptible phenotypic nature of the isolates. High prevalence of VIM-2, NDM-1, and OXA-23 genes was observed in *A. baumannii* isolates. Interestingly, NDM-1 and OXA-58 were traced in 10 and3 *A. baumannii* isolates respectively; 13 of 22 (59%) isolates showed novel Sequence Types (STs) in the Multi-Locus Sequence Typing (MLST) analysis. ST 1087 was most commonly found ST among all others (16 STs). Conclusions: This study indicated a possible role of *carO* porin genes and adeG (RND) andabeM (MATE) efflux pumps in carbapenem susceptibility of *A. baumannii*. New STs were also reported in the majority of the isolates.

## 1. Introduction

Carbapenem-resistant *Acinetobacter baumannii* has recently surfaced as a significant nosocomial bug. According to the recent reports, carbapenem resistance in *A. baumannii* poses a significant threat to public health [1]. *A. baumannii* commonly causes healthcare-associated infections (HAIs), particularly in intensive care units (ICUs) where the incidence has rampantly escalated over time. Multidrug-resistant (MDR) isolates of *A. baumannii* have been associated with a medical history of long hospitalization, patients on mechanical ventilation, presence of catheters, and with immunocompromised or severely ill patients [2]. *A. baumannii* is known to cause severe life-threatening infections, including meningitis, pneumonia, and sepsis [3]. Compared with resistant ones, carbapenem-susceptible *Acinetobacter baumannii* (CSAB) has gained lesser attention in pathogenesis [4]. Recently, it has been reported that CSAB isolates survived the antibiotic stress through their ability to from biofilm [5]. *A. baumannii* has acquired resistance to almost all the commonly used antimicrobials, which include quinolones, aminoglycosides, and broad-spectrum β-lactams [6]. Multidrug-resistant strains of *A. baumannii* have become a common problem in hospital settings [7]. While carbapenems are considered as antibiotics of last resort for the treatment of *A. baumannii*, the resistance rates are drastically increasing against these antimicrobials worldwide [8]. Carbapenem resistance in *A. baumannii* is conferred by the coexistence of several mechanisms, including decreased permeability of the outer membrane through porins and escalated expression of efflux pumps and the presence of beta-lactamases [9]. Efflux systems of bacteria pump out not only the antibiotics but also several other metabolites and add on to the pathogenicity of *A. baumannii* [10].

The RND (Resistance Nodulation Cell Division) efflux superfamily is most frequently associated with antimicrobial resistance in various gram-negative bacteria, including *A. baumannii* [11,12]. This efflux pump is composed of a cytoplasmic membrane-spanning transporter protein interacting with specific protein (OMP) located on the outer membrane and a periplasmic protein MFP (membrane fusion protein). Up-regulation of efflux pump expression has led to a rise in antimicrobial resistance in *A. baumannii*. Along with the RND family, MATE (multidrug and toxic compound extrusion) has also been observed as contributing to escalated resistance in *A. baumannii* [12]. Screening of such efflux genes has previously been reported in genetically diverse, predominantly European *A. baumannii* isolates.

Resistance to carbapenems in *A. baumannii* is also conferred by carbapenem-hydrolyzing enzymes [13,14]. The first report of the rising of carbapenem resistance was encountered in 1993, followed by periodic episodes from India and all over the globe [15]. Genes encoding different types of beta-lactamases have been detected in *A. baumannii*, which hydrolyze carbapenems, i.e., class-D (blaOXA-23-like, blaOXA-51-like, blaOXA-24-like or blaOXA-40-like, blaOXA-58-like, blaOXA-235-like, andblaOXA-143-like) and class-B beta-lactamases (NDM-1, VIM-1, SIM-1, and IMP). In *A. baumannii*, class-D beta-lactamases have been reported as the most commonly distributed carbapenemases [16]. In one of our previous studies, blaOXA-23-like appeared to be the most prevalent one in the CRAB [17].

Recently, survival of CSAB in the presence of antibiotic stress has been reported, which questioned the role of efflux pumps, porins and beta-lactamases in the augmentation of resistance in the latter. Hence, the present study was performed to screen the selected carbapenemases, efflux transporters, and porins using PCR. Further, quantitative real-time gene expressions of RND (AdeABC, AdeFGH, AdeIJK, and AdeXYZ), MATE (AbeM) efflux pumps, and porins (*carO* and *oprD*) were investigated and correlated with the susceptibility pattern of CSAB. MLST was performed to observe the clonal relationship and genetic relatedness of all the isolates.

## 2. Materials and Methods

### 2.1. Bacterial Isolates

A total of 30 MDR *A. baumannii* isolates were collected from Nehru Hospital, Post Graduate Institute of Medical Education and Research (PGIMER), and Government Medical College & Hospital (GMCH) Chandigarh, India. Bacterial strains were isolated from different specimens, including blood, sterile body fluids (BF), and cerebrospinal fluids (CSF). Specimens were cultured on MacConkey and blood agar plates followed by overnight incubation at 37 °C. Pure colonies were identified by MALDI-TOF-MS (Matrix-assisted laser desorption ionization-time of flight mass spectrometry) [18]. Species identification was confirmed by detection of the OXA-51 gene and *gyrB* multiplex PCR.

### 2.2. Drug Susceptibility Profile

Antimicrobial susceptibility was determined for *A. baumannii* isolates for the following antibiotics using the Kirby-Bauer method: ceftazidime (CAZ), cefepime (FEP), cefotaxime (CTX), amikacin (AMK), chloramphenicol (CHL), piperacillin-tazobactam (TZP), imipenem (IPM), meropenem (MEM), and doripenem (DOR). The MICs (minimum inhibitory concentrations) of imipenem, meropenem, doripenem, and colistin (CST) were calculated by performing an agar dilution method for the CSAB isolates (Table 1). The susceptibility data were documented as resistant (R), susceptible (S), or intermediate (I). The CLSI guidelines were followed to interpret the susceptibility results according to the manual’s instructions [19].

### 2.3. Carbapenemases Screening by PCR

Major groups of carbapenemases, including class-D (OXA-23, OXA-58, OXA-24) and Class-B (NDM-1, VIM, SIM & IMP) beta-lactamases were detected by PCR along with the intrinsically encoded gene OXA-51. PCR was performed using ready-to-use PCR master mix (Sigma Aldrich, St. Louis, MO, USA) in the ‘Veriti’ PCR machine (Applied Biosystems, Foster City, CA, USA).

### 2.4. Detection of Efflux Pumps and Porin Genes

The detection of genes encoding the efflux pumps (*AdeB, AdeG, AdeJ, AdeY*, and *AbeM*) and porins (*oprD* and *carO*) was investigated by PCR. Primers are listed in Supplementary Table S1. PCRs were performed containing 2.5 mM dNTP mixture (Sigma Aldrich), 1 unit (0.2 µL) of Taq Polymerase (Sigma Aldrich), 10 pmol of each primer, and 1 µL of bacterial DNA in a final reaction volume of 25 µL. The following parameters were used for thermal cycling: DNA denaturation at 95 °C for 5 min, 35 cycles of 95 °C for 30 s, amplification at 55–60 °C for 30 s, extension at 72 °C for 1 min, and final elongation at 72 °C for 5 min. PCR products were run on agarose gel electrophoresis, stained with EtBr (ethidium bromide), and gel images were captured after visualization on a UV transilluminator.

### 2.5. RNA Isolation & cDNA Preparation

The bacterial isolates were grown at a sub-inhibitory concentration of imipenem (6 µg/mL) overnight at 37 °C on MHA plates, and the total RNA of cells was isolated from late log-phase cultures by a High Pure RNA Isolation Kit (Roche, Mannheim, Germany), as directed by the manufacturer. Absence/presence of contaminating DNA in the samples was observed using controls without RTase (reverse transcriptase). cDNA was prepared from template RNA using reverse transcriptase and random hexamer primers provided in the kit (Roche, Mannheim, Germany). The cDNA was stored at −20 °C. Prepared cDNA was used to perform the downstream RT-PCR amplification.

### 2.6. RT-PCR

Real-Time PCR (RT-PCR) was used to analyze the expression of efflux pumps (*adeB, adeJ, adeG, adeY*, and *abeM*) and *oprD* and *carO* (porins or outer membrane proteins) genes in all the 30 isolates under study. Expression of mRNA was investigated for the efflux pumps and porins genes (Supplementary Table S1) using SYBR Green I (Roche, Mannheim, Germany) chemistry according to the manufacturers’ instructions. Each PCR tube (200 µL) had a reaction mixture of following components: 1 pM of each primer; 1 µL of template cDNA (100 ng/µL); 5 µL of Light Cycler 480 SYBR Green I (Roche, Mannheim, Germany) master mix; and 2 µL of nuclease-free water (Hi-Media Laboratories GmbH) in a final volume of 10 µL. PCR was performed for the prepared reactions following the PCR conditions: 1 cycle of 94 °C for 5 min; and 40 cycles of 94 °C for 20 s, 60 °C for 20 s, and 72 °C for 30 s. Analysis for the melt curve was also performed to ensure production of single amplicon after each run. *A. baumannii* ATCC 19606 and ‘RNA polymerase sigma factor’ gene (*rpoD*) were used as a reference strain and an internal control gene respectively. All reactions were performed in triplicate and the mean CT value was utilized to analyze the expression level for each isolate.

### 2.7. Analysis of Gene Expression

The 2^−∆∆CT^ method was used to calculate the relative gene expression [20]. The *RpoD* (housekeeping) gene was utilized as an endogenous control and calibrated relative to a carbapenem-susceptible reference strain (ATCC 19606) for normalization of expression. An RQ (Relative Quantification) value equal to 1 indicates the similar expression level of the tested gene in both the reference and test (Supplementary Table S2). Increases or decreases in gene expression (Log RQ values) of >2 and <0.5 values were taken as significant expressions respectively.

### 2.8. Multi-Locus Sequence Typing

A total of 22 isolates of *A. baumannii* were included for MLST typing (Oxford Scheme) using seven housekeeping genes (*gltA, gyrB, gdhB, recA, cpn60, gpi*, and *rpoD*) as reported earlier [21]. PCR were performed using the same PCR ingredients as used previously in the PCR based detection of carbapenemases and efflux pumps. Standard PCR profiles and primers were used to amplify DNA of 22 isolates using all seven housekeeping genes of the oxford scheme as performed previously [21]. PCR products were eluted, purified, and sent for DNA sequencing. Resulting sequences of all the genes were analyzed by DNA Star and Sequence Types (STs) were retrieved from the PubMLST (https://pubmlst.org/, accessed on 9 February 2022) database. New combination sequences were assigned as ‘new allele’ numbers followed by new STs using the PubMLST database (https://pubmlst.org/, accessed on 9 February 2022). A phylogenetic tree was constructed to observe the genetic relatedness. The evolutionary history was inferred by the Maximum Likelihood method based on the Tamura-Nei model [22]. The tree with the highest log likelihood (−5805.4662) is plotted. Initial tree(s) for the heuristic search were obtained automatically by applying Neighbor-Join and BioNJ algorithms to a matrix of pairwise distances estimated using the Maximum Composite Likelihood (MCL) approach, and then selecting the topology with the superior log likelihood value. The tree was drawn to scale, with branch lengths measured in the number of substitutions per site. The analysis involved 22 nucleotide sequences. Codon positions included were 1st + 2nd + 3rd + noncoding. All positions containing gaps and missing data were eliminated. There were a total of 2895 positions in the final dataset. Evolutionary analyses were conducted in MEGA6 [23].

## 3. Results

### 3.1. Isolates Description

Overall, 30 CSAB isolates were included in the present study, which were collected from 12 different wards/ICUs/centers of a tertiary care hospital and from three sources: blood, CSF, and sterile body fluid. Most of the specimens were received from Advanced Paediatrics Centre (*n* = 10, 66%), followed by Emergency (*n* = 6, 20%) and Advanced Trauma Centre (*n* = 3, 10%).

### 3.2. Antimicrobial Susceptibility Testing

All 30 CSAB isolates were initially identified by MALDI-TOF MS followed by confirmatory OXA-51 PCR. All the isolates were susceptible to imipenem, meropenem, doripenem, and colistin as confirmed by MIC (Table 1). All the isolates except a few showed resistance to the six tested antimicrobials by Kirby Bauer disk diffusion assay (Table 1).

### 3.3. Carbapenemases Detection

**Figure 1:**
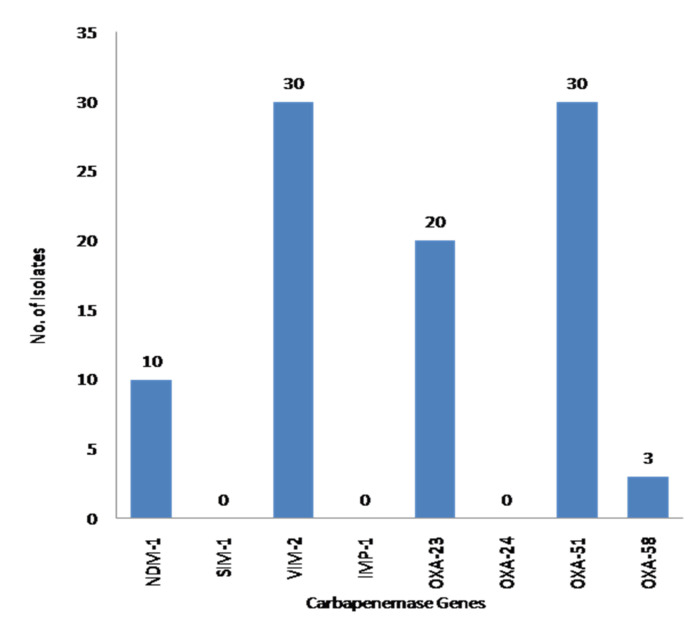
Prevalence of carbapenemases in the clinical isolates of *A. baumannii*.

All CSAB isolates showed presence of OXA-51 and VIM-2 carbapenemases genes. Most of the isolates were found harboring OXA-23 (*n* = 20, 66%) (Table 1, Figure 1). NDM-1 was present in 10 CSAB isolates (33%), whereas OXA-58 was present in only three CSAB isolates (Table 1, Figure 1). Interestingly, three isolates co-harbored NDM-1 and OXA-58, out of which two showed new STs (1089), isolated from CSF at Advanced Trauma Centre (Table 1, Figure 1). SIM-1, IMP-1, and OXA-24 were not found in any of the CSAB isolates (Table 1, Figure 1).

### 3.4. Efflux Pump and Porin Genes Expressions

**Figure 2:**
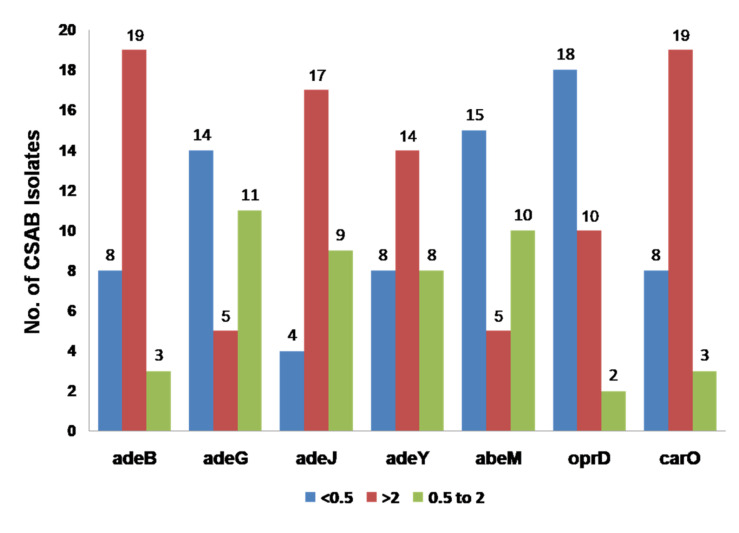
Expression of RND-efflux pumps and outer membrane porins in *A. baumannii* isolates. Values of >2 showed over-expression, <0.5 showed under-expression, and 0.5–2.0 showed no significant expressions.

In the present study, *adeB, adeJ*, and *adeY* showed over-expression (>2.0 fold) in 19 (63%), 17 (57%), and14 (47%) CSAB isolates respectively (Figure 2, Table 1) and were found non-significant pertaining to CSAB. On the other hand, *adeG* and *abeM* showed under-expression (<0.5 fold) in 14 and 15 (48% and 50%) CSAB isolates respectively. Likewise, the *carO* porin gene showed over-expression in 19 (63%) isolates, which cumulatively suggested the susceptible phenotype of the isolates (Figure 2, Table 1). *AdeB* and *adeY* under-expressions were reported in eight (27%) CSAB and *adeJ* accounted for four (13%) isolates; 10 (33%) CSAB isolates showed over-expression of *oprD* and only 18 (60%) isolates showed under-expression of the latter (Figure 2, Table 1, Supplementary Table S2).

### 3.5. MLST Analysis

**Figure 3:**
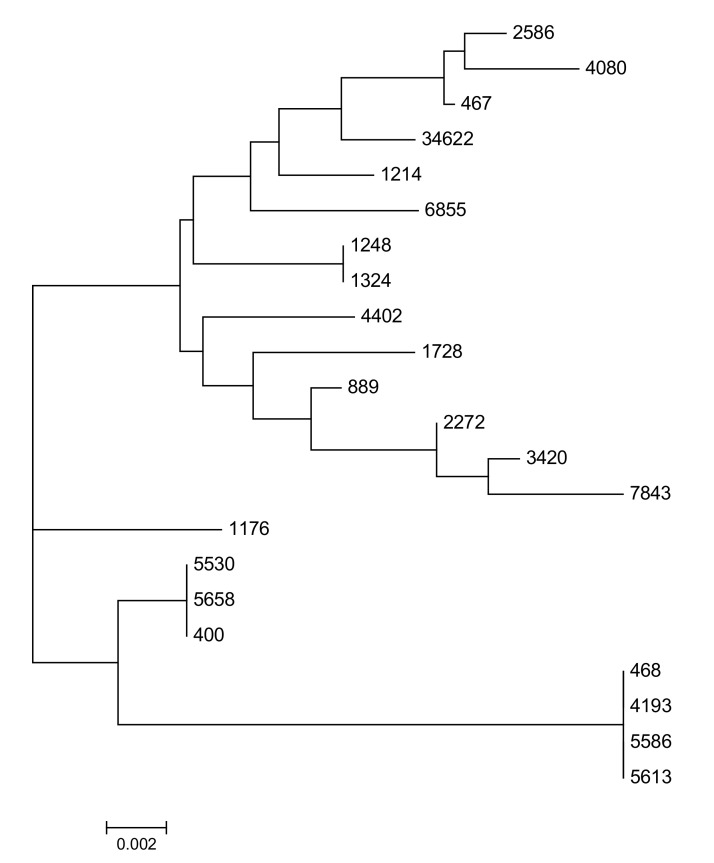
Molecular Phylogenetic tree of 22 CSAB isolates constructed by the Maximum Likelihood method using 6th version of MEGA software.

A total of 16 STs were identified in 22 CSAB isolates. MLST analysis showed 13 of 22 (59%) isolates with novel STs (Table 1). Out of 16 isolates with new ST type, six were harboring OXA-23 (38%), whereas two isolates co-harbored NDM-1 and OXA-58. Nine isolates showed the previously reported seven STs (not novel), in which ST-447 was the most common (*n* = 3). One isolate with ST441 represented the international clonal complex I (CC-I) and another isolate with ST 451 represented the CC-II (Table 1). This revealed that CSAB isolates with novel STs showed high genetic changes but did not fall under internationally circulating clones. Molecular phylogenetic analysis using maximum likelihood tree showed diversity in all the isolates of CSAB. Two major lineages were seen in the phylogenetic analysis where one was bigger, harboring 14 CSAB isolates while the other, smaller one harbored7 CSAB (Figure 3). Surprisingly, one isolate (1176) was seen in solitary and was not included in any of the two major lineages (Figure 3). Whole genome sequencing (WGS) could discern the fundamental reason behind the distribution of these isolates into different lineages.

## 4. Discussion

RND-type efflux pumps are encoded by chromosomal genes and their over-expression contributes to multidrug resistance in *A. baumannii* [24]. In the present study, four RND efflux pumps were investigated in *A. baumannii* clinical isolates: AdeABC, AdeIJK, AdeFGH, and AdeXYZ. Among these, AdeABC is considered as the significant one associated with multidrug resistance including carbapenems [25]. Efflux transporters *adeG* and *abeM* showed under-expression (<0.5 fold) in 14 and15 (50%) CSAB isolates respectively, along with *carO* porin genes, which showed over-expression in 19 (63%) isolates, cumulatively suggesting the carbapenem-susceptible phenotype of the isolates (Figure 2, Table 1). The overexpression of the *carO* gene seems to be crucial for retention of the carbapenem in the bacterium and rendering it to be susceptible. A bacterium is considered to be susceptible if it overexpresses the porin genes and under expresses the efflux pump genes, which in turn enable the antimicrobial to retain inside the bacterial cell for a prolonged period of time. This pattern was noticed in the present study for the *carO* porin gene and *adeG* and *abeM* efflux pump genes (Figure 2, Table 1). As per recent reports, AdeFGH over-expression is found to confer multidrug resistance in *A. baumannii* isolates [26,27]. *OprD* gene also showed over-expression, but only in the 10 (33%) isolates. *AdeY* shared >97% homology with *adeJ*, which has already been identified in the Acinetobacter strain and suggested a probable involvement in the intrinsic resistance to β-lactams, tetracycline, ciprofloxacin, chloramphenicol, and rifampin [28,29]. Efflux pumps *adeB, adeJ*, and *adeY* usually over express in CRAB isolates but in the present study, surprisingly, they showed overexpression in 19 (63%), 17 (57%), and 14 (47) CSAB isolates. *AdeB, adeJ*, and *adeY* showed under-expressions but that was seen only in eight (27%), four (13%), and eight (27%) isolates respectively. This suggested that overexpression of the latter is probably associated with antimicrobials rather other than carbapenems but this hypothesis requires further investigation. *AbeM* belongs to the MATE super family, which is an H^+^-coupled multidrug efflux pump and known to extrude fluoroquinolones, aminoglycosides, chloramphenicol, ethidium bromide, and trimethoprim in *A. baumannii* [30]. Previously, *abeM* was found in clinical isolates of *A. baumannii* and tested without any correlation with antibiotics even when it showed overexpression [31,32]. In the present study, 15 (50%) of the CSAB isolates showed under-expression of *abeM,* suggesting its involvement in carbapenem susceptibility.

*OprD* plays a critical role in conferring resistance to gram-negative bacilli. The presence of *oprD* in lower numbers or its downregulation adds on to the resistant phenotype. In a study, mutations in *oprD* were found as associated with imipenem resistance in *A. baumannii* [33]. In the current study, 10 (33%) CSAB isolates showed over-expression of *OprD* and 18 (60%) isolates showed under-expression, thereby showing a meager association with susceptible phenotypes (Figure 2, Table 1). In a previous study, the RT-PCR was used to correlate the expression of porin (*carO, oprD*)-coding genes with the antimicrobial resistance of *A. baumannii* [34] and a significant association was observed with decreased expression of porins in resistant isolates. On the other hand, interestingly, 19 (63%) CSAB isolates showed over-expression of the *carO* gene and hence rendered the *A. baumannii* isolates susceptible (Figure 2, Table 1).

To date, most of the studies have suggested a crucial role of OXAs in conferring carbapenem resistance to *A. baumannii* [35]. The present study also revealed high prevalence of OXA-23 in the CSAB isolates (Figure 1). However, the contribution of NDM-1 in the development of carbapenem resistance cannot be ignored in Indian scenario. Contrary to recent studies from India, which have identified the significant contribution of NDM-1 in carbapenem resistance, in the present study, 10 (33%) CSAB isolates showed the presence of the NDM-1 gene (Figure 1) [36]. In another study, we already detected NDM-1 in 29.7% of CRAB isolates [17]. However, a few carbapenamses, including OXA-24, IMP-1, and SIM-1, were not detected in any of the CSAB isolates (Figure 1). MLST analysis showed 13 of 22 (59%) isolates with novel STs (Table 1). A total of 16 STs were identified and assigned to 22 CSAB isolates, of which ST 1087 (4N) was the most commonly found ST, followed by ST 447 (3N) and ST 1089 (2N) (Table 1).

## 5. Conclusions

To summarize, this study showed significant findings, including over-expression of *carO* and significant down-regulation of *adeG* (RND) and *abeM* (MATE) efflux-pumps, which contribute to the carbepenem-susceptible phenotypes. Co-existence of NDM-1, OXA-23, and OXA-58 genes was also observed in a few isolates. Further, WGS-based genomic analysis of the regulatory network of efflux pumps and porins could better reveal the fundamental reason of antibiotic resistance in CSAB isolates. Relatively many new STs were reported in CSAB isolates, which suggested a high level of recombination in the genomes of CSAB isolates. WGS can be used to surface the altering molecular pattern and structures of old antibiotics and their interactions with efflux pump components, which could be a promising strategy to control the efflux pump expression and thereby the escalating antimicrobial resistance can be curbed.

## Figures and Tables

**Table 1:** Antimicrobial susceptibility testing, RT-PCR gene expression, Carbapenmases, and MLST sequence types of CSAB isolates. Abbreviations of Antimicrobial Agents: IPM—Imipenem; MEM—Meropenem; DOR—Doripenem; CST—Colistin; CHL—Chloramphenicol; AMK—Amikacin; CAZ—Ceftazidime; FEP—Cefepime; CTX—Cefotaxime; TZP—Piperacillin-Tazobactam. Abbreviations of Wards: EMG—Emergency; MICU—Main ICU; APC—Advanced Paediatrics Centre; BICU—Burn ICU; MSW—Male Surgical Ward; ATC—Advanced Trauma Centre; SLR: Septic Labour Room; NICU—Neonatal ICU; AGE—Advanced Gastroenterology Centre; CTVS—Cardiovascular & Thoracic Surgery Unit; OPD—Outside Patient Department; EMW—Emergency Ward.

S. No.	Lab No.	Sources	Gender	Wards	MIC Values				DD Assay						Carbapenemases								MLST	
					IPM	MEM	DOR	CST	CHL	AMK	CAZ	FEP	CTX	TZP	NDM-1	SIM-1	VIM-2	IMP-1	OXA-23	OXA-24	OXA-51	OXA-58	ST	ST Type
1	34,622	Blood	M	EMG	S	S	S	S	R	R	R	R	R	R	+	-	+	-	-	-	+	+	930	-
2	2586	Blood	M	MICU	S	S	S	S	R	R	R	R	R	R	+	-	+	-	+	-	+	-	539	-
3	400	BF	M	APC	S	S	S	S	R	R	R	R	R	R	-	-	+	-	+	-	+	-	447	-
4	2272	Blood	F	BICU	S	S	S	S	R	S	R	R	R	R	-	-	+	-	+	-	+	-	1086	New
5	467	BF	M	MSW	S	S	S	S	R	R	R	R	R	R	-	-	+	-	+	-	+	-	451	-
6	468	BF	F	EMG	S	S	S	S	R	R	R	R	R	R	-	-	+	-	+	-	+	-	1087	New
7	3420	Blood	M	APC	S	S	S	S	R	S	S	R	R	S	-	-	+	-	-	-	+	-	1088	New
8	1176	CSF	F	EMG	S	S	S	S	R	R	R	R	R	R	+	-	+	-	+	-	+	-	441	-
9	1214	CSF	M	ATC	S	S	S	S	R	R	R	R	R	R	-	-	+	-	-	-	+	-	1112	New
10	4080	Blood	F	SLR	S	S	S	S	R	R	R	R	R	R	-	-	+	-	+	-	+	-	195	-
11	4193	Blood	M	NICU	S	S	S	S	R	R	R	R	R	R	-	-	+	-	+	-	+	-	1087	New
12	1248	CSF	M	ATC	S	S	S	S	R	R	R	R	R	R	+	-	+	-	-	-	+	+	1089	New
13	4402	Blood	F	AGE	S	S	S	S	R	R	R	R	R	R	-	-	+	-	+	-	+	-	860	New
14	889	BF	M	CTVS	S	S	S	S	R	R	R	R	R	R	-	-	+	-	+	-	+	-	387	-
15	1324	CSF	M	ATC	S	S	S	S	R	S	R	R	R	R	+	-	+	-	-	-	+	+	1089	New
16	1728	BF	M	OPD	S	S	S	S	R	S	R	R	R	S	-	-	+	-	-	-	+	-	1393	New
17	5530	Blood	M	APC	S	S	S	S	R	R	R	R	R	R	-	-	+	-	+	-	+	-	447	-
18	5586	Blood	M	APC	S	S	S	S	R	R	R	R	R	R	-	-	+	-	+	-	+	-	1087	New
19	5613	Blood	M	EMG	S	S	S	S	R	R	R	R	R	R	-	-	+	-	+	-	+	-	1087	New
20	5658	Blood	M	APC	S	S	S	S	R	R	R	R	R	R	-	-	+	-	+	-	+	-	447	-
21	5869	Blood	M	APC	S	S	S	S	R	R	R	R	R	R	-	-	+	-	+	-	+	-	NA	-
22	6131	Blood	M	EMG	S	S	S	S	R	R	R	R	R	R	+	-	+	-	+	-	+	-	NA	-
23	6173	Blood	M	APC	S	S	S	S	R	R	R	R	R	R	+	-	+	-	+	-	+	-	NA	-
24	1818	CSF	F	EMG	S	S	S	S	R	R	R	R	R	R	+	-	+	-	-	-	+	-	NA	-
25	6266	Blood	M	BICU	S	S	S	S	R	R	R	R	R	R	+	-	+	-	+	-	+	-	NA	-
26	6826	Blood	M	CTVS	S	S	S	S	R	R	R	R	R	R	-	-	+	-	+	-	+	-	NA	-
27	6855	Blood	M	APC	S	S	S	S	R	S	R	R	S	R	-	-	+	-	-	-	+	-	1394	New
28	7021	Blood	M	APC	S	S	S	S	R	S	R	R	R	S	-	-	+	-	-	-	+	-	NA	-
29	7435	Blood	F	EMW	S	S	S	S	R	R	R	R	R	R	+	-	+	-	+	-	+	-	NA	-
30	7843	Blood	F	APC	S	S	S	S	R	S	R	S	S	S	-	-	+	-	-	-	+	-	1090	New

## Data Availability

Not applicable.

## Supplementary Materials

The following supporting information can be downloaded at: https://www.mdpi.com/article/10.3390/life12030419/s1, Table S1: List of Primers used for RT-PCR; Table S2: Antimicrobial susceptibility testing, RT-PCR gene expression, Carbapenmases and MLST sequence types of CSAB isolates.
