# Supplementary material for: Differential Gene Expression of Efflux Pumps and Porins in Clinical Isolates of MDR Acinetobacter baumannii
Source: Life (Basel). 2022 Mar 14;12(3):419. doi: 10.3390/life12030419 (PMC8948634; doi:10.3390/life12030419)
Supplement: Supplementary file 1 [file life-12-00419-s001.zip › life-1612875-supplementary.pdf]

life

MDPI

Supplementary Materials: Differential Gene Expression of Efflux Pumps and Porins in Clinical Isolates of MDR *Acinetobacter baumannii*

Table S1. List of Primers used for RT-PCR.

S.No.	Gene	Sequence (5′---3′)	Annealing Temperature
1	rpoD-F	ACCCGTGAAGGTGAAATCAG	60°C
	rpoD-R	TTCAGCTGGAGCTTTAGCAAT	
2	adeB-F	TTAACGATAGCGTTGTAACC	60°C
	adeB-R	TGAGCAGACAATGGAATAGT	
3	adeG-F	ATCGCGTAGTCACCAGAACC	55°C
	adeG-R	CGTAACTATGCGGTGCTCAA	
4	adeJ-F	ATTGCACCACCAACCGTAAC	60°C
	adeJ-R	TAGCTGGATCAAGCCAGATA	
5	adeY-F	AATTGCAGGAAAAAGGTGAA	55°C
	adeY-R	GGAAGTACACCGAAACCGAA	
6	abeM-F	GTAGGTGTAGGCTTATGGA	60°C
	abeM-R	GTACCGAAGTGACTGAAAT	
7	oprD-F	TAAGCTGAACCATCGTTGTG	60°C
	oprD-R	GAGCAAAGTGAGGCAAAAGG	
8	carO-F	GTTATGGCAAGCAAACCCAT	60°C
	carO-R	TGACCAGGAACTGCTTGTTG	

Table S2. Antimicrobial susceptibility testing, RT-PCR gene expression, Carbapenmases and MLST sequence types of CSAB isolates.

Abbreviations of Antimicrobial Agents: IPM – Imipenem; MEM – Meropenem; DOR – Doripenem; CST – Colistin; CHL – Chloramphenicol; AMK – Amikacin; CAZ – Ceftazidime; FEP – Cefepime; CTX – Cefotaxime; TZP – Piperacillin-Tazobactam.

Abbreviations of Wards: EMG - Emergency; MICU – Main ICU; APC – Advanced Paediatrics Centre; BICU – Burn ICU; MSW – Male Surgical Ward; ATC – Advanced Trauma Centre; SLR: Septic Labour Room; NICU – Neonatal ICU; AGE – Advanced Gastroenterology Centre; CTVS – Cardiovascular & Thoracic Surgery Unit; OPD – Outside Patient Department; EMW – Emergency Ward.

S. No.	Lab No.	Sources	Gender	Wards	MIC Values				DD Assay (Zone Diameter)						Efflux Pumps Expression (2-ΔΔCT Method based Log RQ values)					Porins Expression		Carbapenemases								MLST	
					IPM	MEM	DOR	CST	CHL	AMK	CAZ	FEP	CTX	TZP	adeB	adeG	adeJ	adeY	abeM	oprD	carO	NDM-1	SIM-1	VIM-2	IMP-1	OXA-23	OXA-24	OXA-51	OXA-58	ST	ST Type
1	34622	Blood	M	EMG	2	1	1	2	12	14	14	13	12	16	-2	1.1	-0	0.1	1.1	0	0	+	-	+	-	-	-	+	+	930	-
2	2586	Blood	M	MICU	1	0.5	0.5	0.5	12	12	13	12	13	17	0	-1	-1	-1	-0.6	-1	1.4	+	-	+	-	+	-	+	-	539	-
3	400	BF	M	APC	2	1	1	1	13	14	14	13	13	16	0	0.1	0.2	0.7	0.1	2	-2	-	-	+	-	+	-	+	-	447	-
4	2272	Blood	F	BICU	1	0.5	0.25	1	12	18	12	12	14	15	-0	-0	-2	-1	-0.4	1.1	-6	-	-	+	-	+	-	+	-	1086	New
5	467	BF	M	MSW	2	2	1	0.5	11	14	12	12	12	16	-2	-0	-0	-0	-0.3	-1	0.2	-	-	+	-	+	-	+	-	451	-
6	468	BF	F	EMG	1	0.5	1	0.5	11	12	14	14	12	16	0	-0	-0	0.1	-0.4	0	-1	-	-	+	-	+	-	+	-	1087	New
7	3420	Blood	M	APC	1	0.25	0.25	0.5	11	17	18	14	13	22	0	0.1	-1	-1	-0.1	0.7	-2	-	-	+	-	-	-	+	-	1088	New
8	1176	CSF	F	EMG	2	1	2	1	12	13	13	13	12	16	-1	1.6	-1	0.2	1.4	-0	-7	+	-	+	-	+	-	+	-	441	-
9	1214	CSF	M	ATC	2	2	2	2	11	12	12	12	13	17	-4	-1	-1	-1	-0.9	-1	-1	-	-	+	-	-	-	+	-	1112	New
10	4080	Blood	F	SLR	1	1	0.5	1	11	11	14	13	13	17	-2	-1	-1	-1	-0.6	0.5	-5	-	-	+	-	+	-	+	-	195	-
11	4193	Blood	M	NICU	0.5	0.5	0.25	1	12	14	14	14	14	17	0	0.6	-1	-0	0.7	0.6	-0	-	-	+	-	+	-	+	-	1087	New
12	1248	CSF	M	ATC	2	2	1	2	12	12	14	12	11	15	-2	-1	-1	-1	-0.8	-2	-1	+	-	+	-	-	-	+	+	1089	New
13	4402	Blood	F	AGE	2	1	0.5	2	12	12	12	12	14	16	-1	-0	-1	-1	-0.3	1.9	-7	-	-	+	-	+	-	+	-	860	New
14	889	BF	M	CTVS	1	0.25	0.25	0.5	11	11	13	12	13	17	-0	-0	-0	-0	-0.5	0	-5	-	-	+	-	+	-	+	-	387	-
15	1324	CSF	M	ATC	1	1	0.5	1	10	17	11	14	11	17	-2	-0	-1	-1	-0.6	0.1	0.9	+	-	+	-	-	-	+	+	1089	New

16	1728	BF	M	OPD	2	2	1	2	10	19	13	12	11	21	-3	-0	-2	-2	-0.3	-4	-7	-	-	+	-	-	-	+	-	1393	New
17	5530	Blood	M	APC	2	0.5	0.25	1	11	13	14	12	12	17	0	0.5	-1	-0	0.8	2.6	-1	-	-	+	-	+	-	+	-	447	-
18	5586	Blood	M	APC	2	2	2	2	12	12	14	12	13	17	-4	-1	-1	-1	-1.1	-3	-1	-	-	+	-	+	-	+	-	1087	New
19	5613	Blood	M	EMG	1	0.25	0.5	0.5	11	14	13	13	14	16	1.6	1.1	-1	0.1	1	0.7	-1	-	-	+	-	+	-	+	-	1087	New
20	5658	Blood	M	APC	1	2	1	0.25	12	12	12	11	13	16	-3	0.1	-1	-1	0.2	-2	-2	-	-	+	-	+	-	+	-	447	-
21	5869	Blood	M	APC	2	0.5	0.5	2	11	12	12	11	13	16	1.5	0.7	-1	-1	0.7	1.9	0	-	-	+	-	+	-	+	-	NA	-
22	6131	Blood	M	EMG	0.5	0.25	0.5	0.25	11	13	13	12	12	17	-2	0.3	-1	-1	0	-2	-1	+	-	+	-	+	-	+	-	NA	-
23	6173	Blood	M	APC	2	2	1	2	12	14	14	11	12	16	-1	-0	-1	-1	-0.3	0.8	-6	+	-	+	-	+	-	+	-	NA	-
24	1818	CSF	F	EMG	1	0.5	1	0.5	11	13	14	14	12	15	-2	-0	-1	-1	-0.4	0.2	-1	+	-	+	-	-	-	+	-	NA	-
25	6266	Blood	M	BICU	2	1	0.5	2	12	13	11	14	12	17	-1	0.2	-0	-0	0.1	1	-6	+	-	+	-	+	-	+	-	NA	-
26	6826	Blood	M	CTVS	0.5	0.5	0.5	0.25	11	12	11	12	14	17	-0	-0	-1	-1	0.1	0.9	-6	-	-	+	-	+	-	+	-	NA	-
27	6855	Blood	M	APC	2	0.5	1	2	11	19	13	12	24	16	-2	-0	-1	-1	-0.1	-3	-0	-	-	+	-	-	-	+	-	1394	New
28	7021	Blood	M	APC	1	1	0.5	0.5	12	18	13	14	14	21	-2	-1	-1	-1	-0.2	-0	-7	-	-	+	-	-	-	+	-	NA	-
29	7435	Blood	F	EMW	2	0.5	0.25	2	12	14	14	12	14	15	-1	0.4	0.3	0.4	0.5	1.3	1.6	+	-	+	-	+	-	+	-	NA	-
30	7843	Blood	F	APC	0.5	0.25	0.5	1	11	17	12	19	23	23	-2	2.3	0.8	1.3	1.9	-2	2.4	-	-	+	-	-	-	+	-	1090	New
